# Unveiling the impact of high-involvement work practices on team creativity: Exploring the interplay of team reflexivity and work demand attributes

**DOI:** 10.1371/journal.pone.0320601

**Published:** 2025-05-05

**Authors:** Abdul Waheed, Zhu Yongyue, Salma Waheed, Shahbaz Hussain

**Affiliations:** 1 School of Management, Jiangsu University, Zhenjiang, China; 2 School of Psychology, Shaanxi Normal University, Yanta District, Xi’an, China; 3 School of Management Science, University of Okara, Okara, Pakistan; National University of Modern Languages, PAKISTAN

## Abstract

This study aims to investigate the association between high-involvement work practices (HIWPs) and team creative performance (TCP) in Pakistan’s hospitality industry. Moreover, the mediating role of team reflexivity (TR) and moderating role of work demand elements workload (WL) and physical work environment (PWE) have also been studied by introducing cognitive workload theory (CWT) in the TCP context. The data were collected from 408 hotel industry employees through a structured questionnaire. The data was analyzed using different statistical techniques including factor analysis, correlation and structure equation modeling, and slope analysis. Results indicate a successful moderation between HIWPs (power, information, and reward) and TR with WL and PWE. Moreover, the results of this study suggest that team reflexivity partly mediates the association between the HIWP and TCP. Results also provide evidence of cognitive workload theory in this study. Considering the results, it is mandatorily recommended to emphasize more on the work demand in the hotel industry. HIWPs should be promoted to increase TCP.

## Introduction

Creativity is one of the significant perspectives that promote valuable and new ideas and support gaining a competitive advantage. In the service industry, especially hotels need creative workers to provide better service quality and to maintain long-term growth [1]. Therefore, considerable attention has been given to exploring the factors influencing creativity, e.g., empowered leadership, employee motivation [2] work environment, and organizational support [3]. Park and Park [4] stated that high-involvement work practices (HIWP) can contribute to employees’ innovative performance. However, most of the earlier studies related to the hotel industry absorbed individual performance outcomes relatively more than team performance. Therefore, it is necessary to evaluate the factors influencing team creative performance in the hotel industry as providing creative products and services in hotels has become essential to meet increasing customer demands and to capture new customers [5]. Hotels are one of the important parts of the service industry and contribute enormously to global economic growth. However, creativity is not triggered only by individuals but it is derived from team efforts [6]. The service-based industries have captured more attention in recent decades, providing more employment opportunities and contributing to countries’ GDP globally. Significant growth in Asian service-based industries is contributing one-third of total output. The service industry has contributed significantly towards the economic development of Asian countries. This significant growth in the service industry encourages investors who have effectively increased the hotels’ infrastructure. Also, this industry got the attention of researchers because it is important for the company’s bottom line [1,7].

Previous literature in the service industry has extensively focused on customer satisfaction and service quality [8]. The hotel industry has a prominent place in the service sector, and many issues related to the hotel industry still need to be addressed [9]. Recently business environment has changed drastically in both developed and developing countries. Countries are trying to shift towards a services-based industry, especially the hotel industry. However, Service-based industries as an integral part of the country’s economy are growing at a slower pace in developing countries such as Pakistan. The service industry of Pakistan contributes 53% of the GDP by the World Factbook which shows that it is a fast-growing industry and a central pillar of success in enhancing the Pakistani economy [10]. However, the growth of the service-based industries is slower in comparison to the manufacturing industry which is due to the lack of competitive HRM practices [11]. This study aims to explore the impact of high-involvement work practices (HIWP) on team creative performance and understand the mechanisms involved. It specifically selected HIWP as an illustrative example of strategic human resource management (SHRM) for several reasons. In the existing literature, there is a tendency to equate HIWS with high-performance work practices (HPWPs) and to use them interchangeably with high-commitment work systems (HCWS). However, all HR practices within HIWP are not necessarily employee-friendly, especially if they are entirely performance-oriented but at the same time not employee-oriented. Notably, limited research on HRM and team creativity suggests that certain combinations of HIWP may not be related to team creativity [12]. Furthermore, it is often assumed that HIWP implies high commitment, and the opposite assumption that higher commitment implies HIWP does not necessarily hold. This distinction arises because higher commitment can be pursued through employment practices alone, without changing job structures, increasing job autonomy, or expanding the scope of initiative [13]. Rather, HIWP represents a coherent, mutually reinforcing, overlapping, and synergistic set of HR practices that emphasize power, information, rewards, and knowledge. These practices help promote employee commitment and involvement [14,15].

HIWP addresses critical employee needs and promotes autonomy, relatedness, and competence, all of which are fundamental to creative performance in teams [16]. Unlike HIWS and HCWS, HIWP places a strong emphasis on a broad range of team-oriented practices [1], which may have a significant impact on team creative performance. Therefore, this study focuses on HIWP to assess its impact on team creativity through the cognitive workload theory (CWT). SHRM scholars believe that HR practices contribute to achieving good organizational outcomes by developing a knowledgeable and motivated workforce that enables them to contribute to the organization’s strategic goals [6,17]. Looking closer at the HRM literature, it is clear that most of the studies focusing on the impact of HIWP are mainly focused on the manufacturing industry [12,18]. However, the presence of other sectors, especially services, has become a critical area that cannot be ignored for two key reasons. The service industry has distinctive characteristics such as “product production and consumption occur simultaneously”, “the intangibility of service processes and results”, and “customers’ participation in service production” [1,19]. Considering the situation, studies linking HIWPs and business performance especially TCP are insufficient [2]. Earlier studies indicated a causal link between HIWPs and TCP as a part of system theory. System theory explains the concept of “Wholeness” where overall system performance depends on subsystems [13]. Industries need desired work results, and the desired results depend on inputs, TCP is a crucial indicator of performance which depends on strong input resources such as HIWPs [20]. Moreover, TCP leads to multiple benefits and it has become a competitive instrument for organizations. Also, Martinaityte, Sacramento [15] explained that competitive tools are an essential part of organizations which determined to explore creative work experiences. But the fact is that organizations are ignoring team creative performance (TCP) surprisingly.

TCP is greatly influenced by grueling work schedules, work-family conflict, and a lack of a supportive learning environment. Previous studies have related TCP with the role of traditional HRM practices[15], high-performance HR practices [21], high-commitment work systems, and team characteristics, e.g., team diversity, team proactivity, team motivation. However, the majority of the studies ignored the importance of HIWPs such as power, reward, information, and knowledge. Moreover, their focus was on the individual level performance, if employees were exposed to different HRM practices, they responded differently due to their characteristics [15]. To become a competitive and productive organization, it is necessary to focus on both individual and team performance. To uncover the mechanism of the relationship between HIWP and team creativity performance, this study demonstrates that team reflexivity is a mediating factor that influences the impact of HIWP on team outcomes. Previous research has shown that team reflexivity is a key mediating factor between HRM practices and their resulting impacts [22]. Team reflexivity plays an important role in members reflecting on their activities and engaging in discussions about goals and strategies. Moreover, the TR concept involves contemplative practices such as exploratory learning, focused attention, self-aware retrospective analysis of past events, and assimilation and integration of new information [23]. Work demands are the pressures experienced by employees in different occupational fields, often in the form of an overwhelming workload within a limited time [24]. This study aimed to investigate how work demand with its attributes moderates the relationship between HIWP and team reflexivity. The association between HIWPs and TCP can be influenced by arbitrating role of team reflexivity (TR) and moderating role of work demand (WD) elements workload (WL) and physical work environment (PWE) which is a hot issue in HRM nowadays, however, rarely discussed in the service industry.

This study makes a significant contribution to the current literature in several ways. First, it extends the existing HRM literature by introducing a multilevel model that examines the impact of HIWP on team creative performance. Drawing on cognitive workload theory (CWT), this study highlights how HIWP promotes and engages employees in team-oriented practices, thereby promoting improved team outcomes and ultimately enhancing team creativity performance. The link between HIWP and team creativity performance is a complex and multifaceted phenomenon that has not been fully explored previously, thereby advancing current understanding of the field. Second, this study delves into important organizational contexts regarding work demands and their characteristics. While an emerging body of research examines work demands and their characteristics as well as team creativity performance in different organizational contexts [7,23], these studies focused on the outcomes of work demands [25]. Comprehensive examinations of work demand characteristics and team creative performance, as well as strategic HR practices included in HIWPs, remain particularly rare. This study therefore contributes to understanding the types of HR practices that are conducive to generating work demands, a key factor influencing team creative performance. Third, by proposing a multilevel model to elucidate the indirect effect between HIWP and team creative performance which is in turn mediated by team reflexivity, this study enriches the existing literature aimed at explaining the transmission paths between HRM and organizational outcomes. This question remains unresolved in the HRM literature, and this study helps fill this gap. Despite the enormous growth in the hotel industry, the traditional human resource practices still prevail and the implications of HIWPs are still limited in the hotel sector of Pakistan.

## Theoretical literature and hypothesis

### Cognitive Workload Theory (CWT)

Cognitive workload theory suggests that the cognitive resources available to individuals are limited and may be overloaded when tasks require excessive mental effort or multitasking. This theory examines the relationship between task demands, cognitive resource capacity, and their impact on performance, attention, decision-making, and learning [7,26]. CWT offers a unique perspective that emphasizes the cognitive demands of tasks and how they are managed in real-time, which differs from the educational focus of cognitive load theory (CLT) and the broader behavioral framework of social cognitive theory (SCT). Disparate CWT focuses on the cognitive demands of tasks, and SCT focuses more on social and environmental influences on learning and behavior whereas CLT is more professional in terms of learning and instruction. In contrast, CWT provides a framework for understanding and managing mental effort during task performance [27,28]. Cognitive workload theory emphasizes that individuals’ cognitive resources are limited. In the context of HIWP, where employees may be involved in a variety of tasks or decision-making processes, it is crucial to understand how cognitive demands influence team creative performance [29]. In the team environment of HIWP, it becomes critical to understand the cognitive workload of each team member. Balancing workload among team members to avoid cognitive fatigue while maximizing creative input is critical to sustained performance. There is currently little conceptualization and empirical research on the potential interactions between HIWP, TR, and TCP. Furthermore, early studies used social exchange theory and the theory of planned behavior to explain the relationship between HIWP and various employee outcomes[16,25]. Few studies have used CWT to explain the relationship between HPWP and employee outcomes. Based on CWT, this article links HIWPs, TR, WD, and TCP, and proposes the latest perspective on the relationship between HIWPs, TR, and TCP.

### High involvement work practices and team creative performance

The fundamental success of the organization is to support employees’ empowerment because employees have great concern with their appraisal, incentives, and rewards system. Therefore, the organization should develop such a scenario in which employees perform their work with high-involvement work practices (HIWPs) and get positive results. Further, organizations have trust in teamwork and create an environment in which employees work creatively. HIWP is characterized by practices such as participatory decision-making, extensive training, and performance-based incentives, which achieve these goals by promoting employee participation, empowerment, and collaboration all of which are key to fostering creative output. Extending the framework’s scalability to different knowledge economy sectors such as IT, finance, or healthcare can further emphasize its adaptability and usability [30,31]. Team creative performance (TCP) constructs a change in organizations and extends the level of competition within the organizational community [10]. Moreover, team creative performance relates to the critical substance of innovative work such as creative measures of work, innovative work ideas, appropriate use of goods, and effective use of services [32]. These essential substances increase the worth of organizational success. The relation between HIWPs and TCP can increase the ability to work successfully in organizations according to CW theory. Researchers suggested that in the human resource department, TCP shows an optimistic effect on HR practices [11,15]. There has been a great debate on whether HRM extends better organizational performance which includes HIWPs [13], HR practices in high performance, and innovation in creative work.

### High involvement work practices and team reflexivity

Team reflexivity is a concept proposed by West [33] and refers to “the degree to which team members collectively reflect on work-related issues, including team goals, strategies, and operating procedures, and adapt to current or anticipated situations.” It involves reflective actions, including exploratory learning, attention, reviewing past events with self-awareness, and digesting and integrating new information [33]. Cognitive workload theory suggests that task demands, cognitive resource capacity, and their impact on performance cues from team managers influence perceptions of acceptable norms of behavior during work [26]. Researchers have empirically shown that HIWPs prompt followers to understand their work environment and encourage certain behaviors in the workplace [22]. Following CW theory, we note that team members recognize cognitive cues provided by HIWPs that demonstrate humility and then form a perception of legitimate development and growth [34]. Such cognitions are conducive to cultivating a reflective atmosphere throughout the team. Specifically, when HIWPs acknowledge the team’s imperfections in past tasks and put achievements in perspective [31], they encourage subordinates to accept their limitations and pursue improvement, legitimizing team members’ self-acceptance and self-transcendence. Hence, we can link the relationship between HIWP and team reflexivity to CW theory.

### High Involvement Work Practices (HIWPs) and Work Demand (WD)

According to the human resource management (HRM) department, the process of HIWPs categories with different other scopes, these scopes classify HIWPs into innovational HRM practices and contingency perception practices independently [1]. A catalog of maintenance development and performance in HR classify system theory. HIWPs further aimed as a part of employees’ improvement in working skills and motivational working performance, and also have four working traits [14]; a) empowerment of employees during the decision-making process; b) interexchange of task information; c) incentive process (e.g., employee would be rewarded in participation, innovative information sharing, positive outcome behavior, and decision making); and d) employees’ skill and essential training [18].

Lateral work demand refers to organized working conditions that are classified and subsequently further divided into two dimensions: workload and physical work environment. De Reuver, Van de Voorde [20] explained work demands are those psychological and physiological job aspects in organizations that reflect the requirement of stable mental and physical conditions related to physical and psychosomatic costs. Thus, we can relate the relationship between HIWP and WD with its elements WL and PWE by CW theory. Therefore, by mental and physical conviction, work demand has a structure that links with employees’ contributions, requirements, roles, expectations, and customs. Employees must be needed to acclimatize or adjust with psychological and physical exertion. Such exertion creates an effect on independent and dependent variables.

### High involvement work practices, team reflexivity, and team creative performance

High-involvement work practices (HPWP) demonstrate the value and importance of employees within the organization and convey a message of organizational commitment and support [20,30]. Combining different HR practices in the form of HPWS has a more significant positive impact on organizational performance than the effects of these practices alone [20]. This research study presents team reflexivity as a proactive activity that serves as a mediator between HIWP and team creative performance. Cognitive work theory is a common academic framework for understanding the causal mechanisms by which HIWP affects organizational outcomes [4]. In work environments characterized by organizational support and resources provided by team leaders, employees develop a sense of psychological commitment to exert additional effort and demonstrate higher levels of organizational dedication and performance. Thus, this collective effect helps to improve firm performance [1]. Conversely, employees facing a dearth of resources encounter greater challenges in performing assigned job responsibilities and are less motivated to engage proactively. Wang, Cui [22] emphasized that collective creativity inside a team is the result of a combination of individual creativity, team composition, team characteristics, team dynamics, and environmental influences. Subsequent research has confirmed the idea that while individual creativity is a fundamental component of team creativity, simple summaries of individual creativity do not directly translate to overall team creativity. Instead, facilitating team creativity requires pertinent team processes and interactions that synergize each team member’s contributions to creativity [19,35]. As an important situational determinant, high-involvement work practice (HIWP) can not only enhance the creativity of individual team members but also have an impact on the entire team process, thereby promoting team creativity. Consistent with this notion, previous empirical studies have effectively established a correlation between team reflexivity and creative performance [36,37].

In teams characterized by increased levels of reflectiveness, members are willing to gather information while performing tasks, draw on their past experiences, and engage in reflective processes throughout task performance [32]. Earlier research has anticipated a mediating function of team reflexivity in bridging team inputs and outputs [22,37]. Working within this dynamic and interactive framework allows team members to not only acquire unique views that are essential to problem-solving and task execution but also to adjust their aims and solutions to changing conditions, which supports the team’s creative performance. Furthermore, the effectiveness of highly reflective teams in fostering innovation goes beyond conflict resolution. Team reflexivity strengthens team creativity by expanding and integrating team members’ cognitive abilities with amassed information and shared insights[37]. Furthermore, as it enhances the team’s strategic direction and harmonizes problem-solving and learning techniques, a positive link is expected between team reflexivity and team creative performance [3,38].

The basis of knowledge, reward, authority, and technology, HIWPs act as a primary feature and generate effects on team performance. Although having the distinctive existence of team performance from HIWPs, organizations increase creativity in work [39,40]. Previous research focused on HIWPs with general team performance such as team working tasks had the same way of fellowship, team command, working environment, process, techniques, and working motive. Even their working awareness and interpretation had similarities. Generally speaking, old research regarding HIWP and team performance had no innovation in their work. Team members have accumulated their planning, interchange working information, and learning perception according to the team’s motivational performance [4,41]. This team’s motivational performance leads to the regulatory procedure of team creative performance (TCP) [7]. Furthermore, researchers suggested that HIWPs can be affected by TCP [6]. Thus, no prior study explained the direct relation between HIWPs, TR, and TCP and their combined role of significance in organizations.

Based on this interconnected principle of HIWPs, the below hypothesis is made;

   H1: HIWP is positively associated with team creative performance.
   H2: HIWP is positively associated with TR.
   H3: TR is positively associated with team creative performance

### Mediating role of team reflexivity

As introduced by Carter and West [36], the level of collaborative reflection on work-related issues among team members is referred to as team reflexivity. It includes the team’s goals, strategy, and operational processes, facilitating adaptation to existing or anticipated circumstances. This concept involves contemplative practices such as exploratory learning, focused attention, self-aware retrospective analysis of past events, and assimilation and integration of new information [23]. In light of organizations’ prioritization of acquiring, developing, and effectively utilizing employee capabilities to enhance performance [22], a substantial body of research underscores the notion that the connection between human resource management (HRM) and employee performance is influenced by factors such as competencies, attitudes, and behavior [41, 42]. Given this context, we propose to incorporate a mediating factor, specifically team reflexivity, to examine its impact on the association between HIWPs and TCP. First, in reaction to environmental changes, team reflexivity entails the joint and explicit assessment of work-related issues, such as team goals, tactics, and procedures [22]. Reflexivity activities encompass a range of cognitive processes such as meticulous observation, acknowledgment, vigilant monitoring, and appraisal of the subject of reflection [36]. The degree of team reflexivity may exhibit significant variation between various teams within an organization and can be impacted by contextual indicators, such as the prevailing corporate culture. Specifically, across several companies that exhibit collectivist cultural norms, employees actively pursue a sense of affiliation and derive satisfaction from their involvement as essential members of a team [43].

Within this particular framework, the establishment of a pleasant and amicable organizational atmosphere, facilitated by the implementation of HIWPs, has the potential to serve as a catalyst for team reflexivity. This is because team members, motivated by a pleasant environment, demonstrate a willingness to stay on the team and actively contribute to the collective outcome. Given the complexity, high demands, and uncertainty of tasks in research teams, their effectiveness depends on external factors such as access to information, resources, and support from the wider environment [44]. An important part of active participation is feeling empowered to question existing issues and express opinions openly. In turn, this plays a decisive role in the generation of innovative concepts and the formulation of fresh proposals [43]. Proponents of CWT argue that positive feedback loops (such as achieving success) caused by team reflexivity can spur teams to set bolder and more innovative goals. This in turn drives team members to commit more resources to achieve these goals [41,44]. Team reflexivity plays a vital role in fostering innovation by facilitating the exchange of ideas about work processes among team members. This allows for the incorporation of different perspectives where necessary and fosters a common understanding that effectively guides team processes and outcomes [42]. This framework supports our proposed hypothesis.

   H4: HIWP’s and TCP’s relationship has partially mediated by team reflexivity.

### Moderating role of work demand

This research has connoted work demand as a moderator between HIWPs and TR. Work demand’s relation with TR is described a bit in Western studies [8]. However, in the context of HIWPs, work demand studied as a meta-analysis, established relations among HIWPs with working hours (22 samples) and supposed workload (10 studies) has significant ties with the workload and working hours. Such findings extended to the Western European country “Netherlands”. Thus on the eastern side, there must be conduction of such working findings [3,20].

In the hotel context, there is an extensive pressure of work on employees, and employees often face conflicting demands from hotels, managers, and customers and these conflicts create tension or dissonance for employees. Investigating the main dimensions of work demands (workload and physical work environment) is important[45]. In this research paper, work demand with its sub-moderator workload and the physical working environment contribute as a moderating role between independent variable HIWPs (power, information, rewards, and knowledge) and TR becomes the dependent variable. The workload as a moderator considers a remarkable between independent and dependent variables. However, researchers didn’t give attention to workload’s moderating role and only believed that workload is psychological pressure. It can be used by non-investigational techniques and objectively can be considered as a particular work behavior in the minimum period. Furthermore, workload is considered an activity that is essential for the work environment [46].

In this investigation workload judges as a technique that affects the intensity of TR and HIWPs in Pakistan’s service industry. In the daily routine hotels, authorities deal with many customers that produce workload. High involvement work practices (HIWPs) cope with this workload and contribute to a beneficial development process which promotes the best customer service in hotel management. Thus, we can link the relationship between HIWP, team reflexivity, and workload to CW theory. Moreover, HIWPs are also helpful for decreasing the workload effects and increasing the team’s creative performance. However, the primary focus of workload in the hotel industry is to enhance the hotel authorities’ work performance and to handle the flow of customers in a limited time span. Thus, the workload can be utilized as an indicator which can be beneficial to treat the flow of customers in the limit zone.

On the basis of the previous researcher’s points of view, the hypothesis is built as under:

   H5a: Workload moderates the relationship among HIWP’s and Team reflexivity.

Mainly physical working environment (PWE) has a portion of the environmental, and psychological field. PWE has been based on the generous worthy working environment which has a generally convenient condition of the workplace. Virtually, this term explains the relations between employees and their PWE. However, researchers related PWE with other factors such as PWE studied with job satisfaction and performance, PWE with employees’ mood, PWE with behavior, and PWE generosity with demand and job stress [47, 48]. Thus, we can link the relationship between HIWP, team reflexivity, and PWE to CW theory.

As a part of the work demand, PWE has many possible challenges. In an organization, workers can complain regarding work environment quality, insolvent environmental acceptance, working limitations, and imitation in reactions and responses in a challenging environment [48]. Also, the quality of PWE can be expected to be an interface between two variables HIWPS and TR. This research studies that if the HIWP condition is high with the combination of low PWE quality, TR would be higher. Consequently, in contrast, it considers that PWE quality can rise high with the intensity of HIWP would be unrelated to the intensity of TR. Hence, based on the research literature, the following hypotheses have been constructed:

   H5b: PWE moderates the relationship among HIWPs and Team reflexivity.

**Fig 1:**
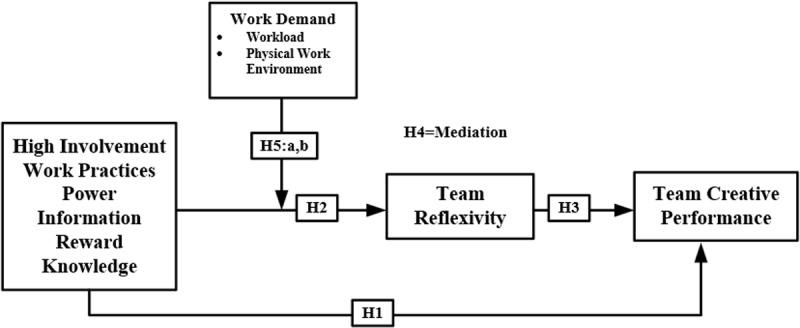
Model of Study.

Based on the above-proposed hypothesis, the study model has been developed as shown in Fig 1 which includes a total of one independent variable HIWPs along with its dimensions (Power, information, reward, and knowledge), one dependent variable (team creative performance), one mediator (team reflexivity) and one moderating variable work demand along with its dimensions (workload, physical work environment). Based on the study model, a total of five hypotheses H1, H2, and H3 have been developed based on the direct relationship between the independent variable HIWPs, intermediary variable TR, and the dependent variable TCP. Then H4 team reflexivity partially mediated among HIWP’s and TCR. Further hypothesis (H5a) presents the moderating role of work demand (workload) between HIWPs and TR. In the last, hypothesis (H5b) describes the moderating relationship of work demand (physical work environment) between HIWPs and TR. Fig 1 illustrates the conceptual framework employed in this investigation, depicting the major variables and corresponding hypotheses.

## Methodology

This research investigates the effect of HIWPs on TCP with the moderating role of work demand attributes and the mediating role of team reflexivity. To achieve the study objective; data were collected from the service industry, more specifically from the hotel sector of Pakistan. This sector is facing many critical issues like low productivity, poor intellectual skills, team performance, and innovation. Two research assistants were from a major university in the twin cities named Islamabad and Rawalpindi Pakistan. The hotels included in the study were not categorized as seven-star or five-star hotels. Instead, they represent a range of midscale and budget accommodations that may have different operating dynamics, service standards, and staff structures than luxury hotels. This distinction is important as it may influence the generalizability of the findings to higher-end hospitality settings and the hotel industry to participate in this survey. The cross-sectional and survey-based data were collected from 660 team members also including 55 team leaders of 30 targeted hotels whose work influenced the team performance. The sample size for this study meets the minimum requirement for analysis, ensuring adequate statistical power and model stability [49, 50]. The decision to include team leaders was intended to capture a broader range of perspectives within the selected hotels. This figure represents the total number of employees across the selected hotels, aimed at ensuring a comprehensive understanding of the workforce dynamics. Earlier studies found that cross-sectional and survey-based data collection are widely used, and cost-effective methods to collect data from a larger population [51]. Further, to solve the issues related to population and sampling, a combination of sampling techniques was utilized [52]. Considering the context of the study, it utilized a purposive sampling technique along with a snowball sampling technique. Initially, the study objective and scope were communicated to the managers, and then it was requested to identify other colleagues, as questionnaires were distributed to team members through their team leaders.

The research has been granted ethical clearance by the Central Academic Review Board (CARB) of the School of Management Sciences at the University of Okara. Informed written consent was obtained from all study participants, who claimed their understanding of the study’s purposes and they would like to participate in the study voluntarily. The respondents were assured that their identities and responses were kept confidential which would be maintained in the best interests of academic integrity and every possible manner. Additionally, they were informed of the importance of the study, its possible implications, their role as participants, and that no compensation would be offered. Respondents were assured they could stop responding and withdraw from the interview at any time. The data collected will be used only to suggest mechanisms for decreasing the impostor’s feelings through academic outcomes. We contacted staff members at different organizational levels, including upper, middle, and front-level staff. This approval allows the research assistants to make a request note in which they express their project work and guarantees the hotel’s authority regarding their privacy—moreover, it confirms the schedule for the official meeting. Both research assistants met with officials including human resource managers, team managers, and supervisors designated from upper-level managers to middle-level supervisors. Both research assistants requested managers to classify their coworkers and survey questionnaires were distributed to subordinates by their team leaders in several departments. A target samples are 660 survey questionnaires dispersed from which 480 responses were received. The final sample after the elimination of missing values was 408 questionnaires, and the further analysis rate was 62%. While this rate may appear lower, it is not uncommon in survey research, particularly in the hospitality sector. In addition, the data collection period is from May 2023 to November 2023.

There were two parts to the questionnaires. One part included demographic questions (Gender, Age, Level of Education, Job Designation, Team Size, and Tenure in the service Industry) and the second part contained key variables of this investigation. Understanding the context is crucial by including detailed demographic data for the current study, which not only increases its credibility but also provides a richer context for interpreting the results. This will allow readers to better understand how the findings vary across different roles, experience levels, and environments within the hospitality industry. A further detailed description of demographic information is seen in Table 1.

**Table 1:** Demographic Respondents Information.

Demographics	Frequency (%)	Demographics	Frequency (%)
**Gender**		**Age**	
Male	258 (63%)	20-35	190(47%)
Female	150(37%)	35-45	127(31%)
		45-65	91(22%)
**Level of Education**		**Job Designation**	
Below under Graduation	132(32%)	Waiter/Maids	175(43%)
Graduate	168(41%)	Team Leader	55(13%)
Post Graduate	105(26%)	Guest Service Supervisor	70(17%)
PhD	3(1%)	Restaurant Supervisors	65(16%)
		Conventional Manager	29(7.40%)
		General Manager	14(3.5%)
**Team Size**		**Tenure in the Services industry**	
5 or below	80(19%)	2-5 years	158(39%)
6–10	112(27%)	6-10years	114(28%)
11–20	123(32%)	7–10 years	90(22%)
21–30	60(14%)	More than 11 Years	46(11%)
More than 31	33(8%)		

## Measures

HIWP as an independent variable was based on power, information, reward, and knowledge which were calculated by using an eight-item scale and team reflexivity (TR) was measured using five-item scales. The measurement of work demand (WD) as a moderator involved the use of an eight-item scale. The dependent variable, TCP, was determined through the utilization of a four-item scale. All procedures used a 5-point Likert-type scale ranging from 1 = strongly disagree to 5 = strongly agree. A further detailed description of demographic information is seen in Table 2 as well as represented in S Table. Details about the measure are given below:

**Table 2:** Description of the variable’s scales.

Variables	Variables Statement	Total items	Source of Items
**High Involvement Work practices**	In an awkward situation, employees have the power to make a decision.	8	**Lawler III, Mohrman [** **14** **]**
	Employees have the opportunity to actively engage in the decision-making process		
	The dissemination of information among personnel		
	The information must be aligned with related job tasks and goals.		
	The reward must be performance-based.		
	The reward is a motivational factor for employees.		
	Knowledge should be extended through a different training plan.		
	Employees should be familiar with rules and regulations.		
**Team Creative Performance**	Team members perform their work originally and practically individually.	4	**Lewis [** **53** **]**
	Each member should be able to generate his creative idea as a team.		
	Each member should have the ability to promote his ideas as a team. Each member should be capable of creating a creative solution to the problem as a team.		
**Workload**	Ability to handle customer care efficiently.	4	**Groenewegen and Hutten [** **45** **]**
	Ability to control a large number of customers.		
	Well, manage customer service hours.		
	Hours for administrative training.		
**Physical Work Environment**	Ability to deal with the incorporating openness of available space.	4	
	Importance of public waiting areas.		
	Availability of facilities.		
	Building conditions that offer services.		
**Team Reflexivity**	Whether the team is working together effectively, would be discussed regularly.	5	**Carter and West (1998)**
	The method adopted to job done will be discussed regularly by the team.		
	Due to changing circumstances, the set objectives can be modified by the team.		
	The feasibility of the set objectives can be reviewed by the team.		
	Information on how well to communicate is often discussed in the team.		

### High involvement work practices

HIWP scale including four practice dimensions from the previous studies we calculated using an eight-item scale developed by Lawler, Lawler III [14]. The HIWP practices include eight items with **α=0.78,** Power (In an awkward situation, employees have the power to make a decision. Employees have the opportunity to actively engage in the decision-making process.), ii) Information (The dissemination of information among personnel. Information must be aligned related jobs task, and goals), iii) Reward (The reward should be based on performance. The provision of rewards serves as a significant motivational component for employees.) and iv) Knowledge (Knowledge should be extended through different training plan. Employees should be familiar with rule and regulation.). All items employed a 5-point Likert-type scale ranging from 1 = strongly disagree to 5 = strongly agree.

### Team creative performance

TCP was measured with a four-item item scale developed by Lewis [54].TCP includes; (four items with **α=0.83**, Team members perform their work originally and practically individually. Each member should be able to generate his creative idea as a team. Each member should have the ability to promote his ideas as a team. Each member should be capable of creating a creative solution to the problem as a team.). Participants indicated each item employed by a 5-point Likert-type scale ranging from 1 = strongly disagree to 5 = strongly agree.

### Work demand

The work demand scale including two practice dimensions from the previous studies was measured using an eight-item scale developed by Groenewegen and Hutten [45]. The two practices include i) Workload (Four items with **α=0.83**, Ability to handle customer care efficiently. Ability to control a large number of customers. Well managed customer service hours. Hours for administrative training.) and ii) Physical work environment (Four items with **α=0.85**,e.g.,Ability to deal the incorporating openness of available space, Importance of public waiting areas, Availability of facilities and Building conditions which offer for services). All items employed a 5-point Likert-type scale ranging from 1 = strongly disagree to 5 = strongly agree.

### Team reflexivity

TR was measured with five scales developed by Carter and West [36]. TR includes; (five items with **α=0.89**, Whether the team is working together effectively, will be discussed regularly. The method adopted to job done will be discussed regularly by the team. Due to changing circumstances, the set objectives can be modified by the team). Participants indicated each item employed by a 5-point Likert-type scale ranging from 1 = strongly disagree to 5 = strongly agree.

### Data analysis

An in-depth understanding of sample characteristics was obtained through descriptive analysis with SPSS 25. The initial evaluation of study measures included evaluating composite reliability (CR), Cronbach’s alpha (α), and average variance extracted (AVE) to ascertain the reliability and validity of the measures. As shown in Table 3, the α value indicates a range of 0.78 to 0.89, and the composite reliability value demonstrates a range of 0.77 to 0.90. These findings suggest that these measures have satisfactory reliability. Furthermore, the range of AVE values observed in this study varied from 0.59 to 0.70, which suggests that the research instrument had convergent validity. These values surpass the acceptable criterion of 0.50 proposed by Fornell and Larcker [55]. To identify the distinctness of study variables, we followed the guidance of Fornell and Larcker [55]. Table 4 demonstrates that for each variable, the square root of the AVE is greater than the correlation of that variable with the other constructs. Variance inflation factors (VIFs) for all predictor variables were below the threshold of 10, indicating that multicollinearity was not a concern in the analysis [49].

**Table 3:** Factors loading and reliability analysis.

Variables	Variables Items	Alpha (α)	CR	AVE
**High Involvement Work practices**	8	0.78	0.77	0.59
**Team Reflexivity**	5	0.89	0.90	0.68
**Work Load**	4	0.83	0.82	0.63
**Physical Work Environment**	4	0.85	0.84	0.65
**Team Creative Performance**	4	0.83	0.81	0.69

**Table 4:** Descriptive statistics and correlation analysis.

Construct	Mean	SD	TR	HIWP	WL	PWE	TCP
**TR**	3.60	0.77	**0.82**				
**HIWP**	3.67	0.80	0.32**	**0.77**			
**WL**	4.02	0.52	0.33**	0.23**	0.79		
**PWE**	3.75	0.56	0.21**	0.27**	0.42**	0.81	
**TCP**	3.80	0.64	0.36**	0.28**	0.19**	0.20 *	**0.83**

Note. N = 408; HIWP=High involvement practices; TR=Team reflexivity; TCP = team creative performance; WD = Workload; PWE=Physical work environment; Diagonal values depict the square root of the Average Variance Extracted; **= p < 0.01; *= p < 0.05.

This provides further evidence that the chosen research metrics are impartial and appropriate for evaluating the prospective model. To ensure the accuracy of self-reported measures, it is recommended to consider the potential impact of common method variance (CMV). The confounding effects of CMV have been shown to exaggerate or deflate the associations between these measures when participants’ answers are affected by social expectations [56].

Consequently, the statistical software AMOS 24 was employed to conduct a sequence of confirmatory factor analyses (CFA) on the study measures using maximum likelihood estimation. Therefore, an analysis was conducted on five sets of fit indices to assess the suitability of fit for the dataset at hand. The relative chi-square (X^2^/df) value of less than 5 suggests a favorable fit of the model [57]. For the Incremental Fit Index (IFI) and the Comparative Fit Index (CFI), values over 0.90 indicate a satisfactory fit [58]. A Root Mean Square Error of Approximation (RMSEA) result that falls below the threshold of 0.08 suggests a satisfactory level of fit for the model [59]. A TLI rating in close proximity to 1 indicates a favorable level of fit [58]. To achieve the goal, a maker-variable test method was employed, as described by Boso, Story [60]. The approach involves conducting a comparative analysis between the suggested model and two alternative competing models to assess the impact of common method variance (CMV) on pre-existing datasets. In the first model, referred known as the method-only model, each item was constrained to load on only a single factor. As can be determined by the values shown (RMSEA = 0.12, CFI = 0.62, TLI = 0.61, IFI = 0.63, X^2^/df = 5.57), the test was underfitted. The next step was to build a second model (a trait-only model) in which each item was given free rein to load the variables that best described them.

The present investigation suggests good model fitting results, RMSEA = 0.08, CFI = 0.92, TLI = 0.89, IFI = 0.90, and Χ^2^/df = 3.77. In a subsequent step, the third model incorporates the assessment of both the method model and the trait model. This entails the incorporation of a shared latent variable into the suggested theoretical framework and establishing connections between this latent variable and all observed elements. The findings indicate that the model fitting effectiveness of model three is superior, as evidenced by the following indices: RMSEA = 0.069, CFI = 0.91, TLI = 0.93, IFI = 0.92, and X^2^/df = 3.43. The results of this study indicate that Model II has a superior level of fit compared to Model I and is more closely aligned with the fit observed in Model III. With the minimal difference in fit indices between models 2 and 3, it can be concluded that the presence of general method bias is not an issue in this dataset. Therefore, this bias is unlikely to distort the interrelationships among the study variables [60].

## Results of hypothesis

### The direct effects and mediation

Hypotheses H1, H2, and H3 propose direct effects. Furthermore, there is an indirect relationship (H4) between highly involved work practices (HIWP) and team creative performance (TCP), mediated by team reflexivity (TR). The evaluation of partial mediation models involved the utilization of the mediation testing approach proposed by Baron and Kenny [61]. This method entails the assessment of mediation models based on three requirements, which necessitate the significance of all direct relationships between variables in the model. The present study’s model suggests that there are substantial direct impacts. Specifically, the relationship between HIWP and TCP (β =  0.28, p <  0.01), HIWP and TR (β =  0.33, p <  0.01), as well as TR and TCP (β =  0.35, p <  0.01) were shown to be statistically significant. Incorporating the mediator (team reflexivity) into the proposed partial mediation model, the connecting HIWP → TR → TCP (β =  0.12, p <  0.05) retained its significance. This suggests partial support for the mediating role of team reflexivity in the relationship between HIWP and team creative performance.

Furthermore, the utilization of bootstrapping with 2000 resampling iterations with replacement and through empirical observation-derived sample distributions (Hayes, 2013) confirmed the persistence of significant indirect pathways (p <  0.01). As well as based on the reduction in path coefficients, shifting from 0.28 to 0.12, alongside the retention of both direct and indirect significantly which can be seen in Tables 5 and 6, the mediation of team reflexivity (TR) between HIWP and team creative performance (TCP), was found to be partial. This supports Hypothesis H3. Moreover, Table 5 results given for the assessment of control variables (age, gender, tenure, and team size) yielded no notable effects significant in the current model.

**Table 5:** Results of hypothesis using path analysis (mediation effect).

Hypothesis	β Values	STDEV	T-values	R^2^ Values	Level of Significance	Mediation Effects
Gender	0.04	0.041	0.025		0.12 *	
Age	0.08	0.064	2.013		0.09 *	
Tenure in Service Industry	0.09	0.012	0.443		0.10 *	
Team Size	0.10	0.054	1.941		0.08 *	
H1: HIWP → TCP	0.28	0.052	6.730	0.24	0.002***	
H2: HIWP → TR	0.32	0.071	3.943	0.28	0.002***	
H3: TR → TCP	0.35	0.065	4.923	0.33	0.002***	
H4: HIWP → TR→TCP	0.12	0.057	2.105	0.36	0.04**	Partial Supported

Note: N = 408; **β**=Path Coefficient; R^2^ = R Squared; HIWP=High involvement practices; TR=Team reflexivity; TCP = team creative performance; *=not significant (p >  0.05); **=p <  0.05; ***= p <  0.01.

**Table 6:** Results of hypothesis using path analysis (moderation effect).

Hypothesis	β Values	STDEV	T-values	R^2^ value	Level of Significance	Moderation Effects
H5:				0.37		
HIWP → TR	0.33	0.053	6.226		0.002***	
H5a: WL → TR	0.25	0.061	4.098		0.003***	Supported
HIWP x WL → TR	0.18	0.091	1.978		0.04**	
HIWP → TR	0.33	0.065	5.076		0.002***	
H5b: PWE→TR	0.19	0.091	2.088		0.04**	
HIWP x PWE → TR	0.13	0.065	2.000		0.03**	

Note: N = 408; **β**=Path Coefficient; R^2^ = R Squared; HIWP=High involvement practices; TR=Team reflexivity; WD = Workload; PWE=Physical work environment; *=not significant (p >  0.05); **=p <  0.05; ***= p <  0.01.

### The moderation effect of work demand

A subsequent set of hypotheses (H5a and H5b) postulated that the correlation between highly involved work practices (HIWP) and team reflexivity (TR) would depend on the moderating effects of workload and physical work environment. To explore this, we initially generated two interactive terms: “HIWP’s workload” and “HIWP’s physical work environment”. These new terms were subsequently incorporated into the proposed model, each term containing its job demand variable. The results showed that the interaction terms “Workload of HIWP” (β =  0.18, p <  0.05) and “Physical work environment of HIWP” (β =  0.13, p <  0.05) showed significance. Based on these results, hypotheses H5a and H5b are empirically supported.

**Fig 2:**
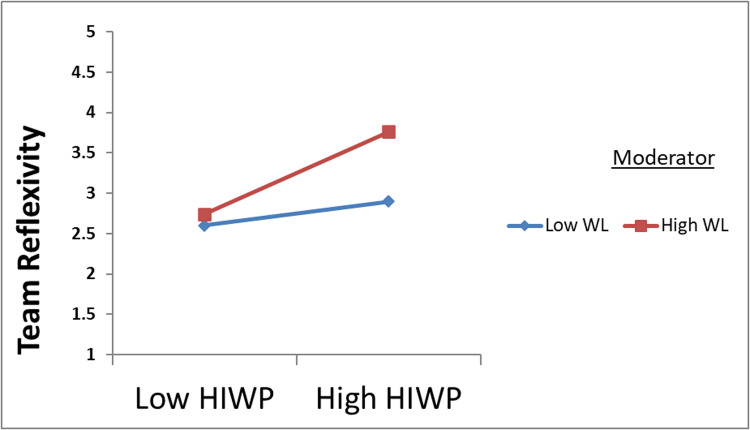
WL strengthens the positive relationship between HIWP and team reflexivity.

**Fig 3:**
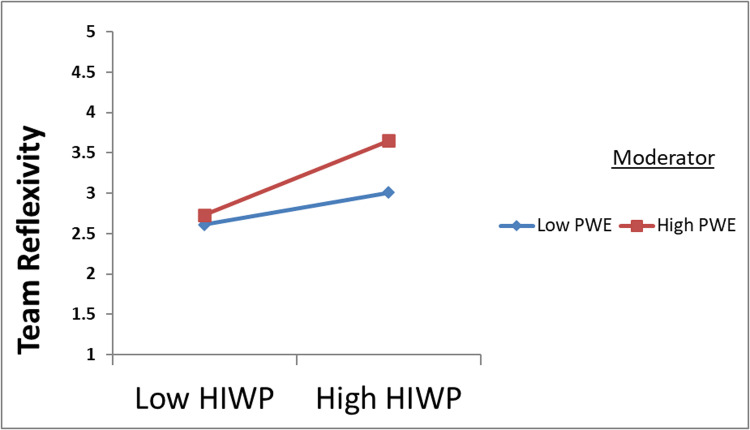
PWE strengthens the positive relationship between HIWP and team reflexivity.

Additionally, an exploration was performed to determine whether the strength of the interactive effects of workload, physical work environment, and HIWP on team reflexivity (TR) differed at low and high levels. To achieve this, operate the low and high levels of each regulator by setting thresholds 1 standard deviation below and above the mean. This approach is consistent with the guidance provided by Preacher, Rucker [62] for assessing statistical significance. As shown in Figs 2 and 3, the results show that the effect of HIWP on TR increases when the workload level increases. Conversely, this effect is attenuated when the workload level is low. Likewise, the effect of HIWP on TR showed greater strength at higher levels of the physical work environment and attenuated at lower levels of the physical work environment.

## Discussion

Through multilevel research, we delve into whether and how organizational-level high-involvement work practices (HIWPs) have an impact on team creativity. Our investigation is consistent with the conceptual analysis, showing a positive association between HIWPs and TCP. In particular, HIWPs demonstrated the ability to create a climate of engagement that facilitates the development of shared leadership within teams, thereby enhancing team creativity performance. Furthermore, this study also raises the idea that team reflexivity (TR) plays a mediating role in the relationship between high-engagement work practice (HIWP) implementation and team creative performance (TCP). Furthermore, we argue that the strength of this relationship is influenced by the specific characteristics of work demands. When examining the mediating process, the results (β =  0.12, p <  0.05) supported a relationship between TCP and HIWP with TR as the mediating mechanism. The explanation of the current study findings can be firmly rooted in cognitive workload theory (CWT). For example, when teams achieve well-structured training programs that support the climate of organization and sustainability goals while improving the workforce’s ability to respond to environmental challenges, teams’ ability to reflect is enhanced [22,25]. Furthermore, when employees actively evaluate and perceive significant alignment between their role performance and clearly defined goals, they tend to actively pursue environmental goals [12,19].

Team relevantly also plays an important role in enhancing team reflexivity. When employees are actively involved in decision-making regarding team learning matters, their intellectual and emotional connections to organizational standards and principles are strengthened. As a result, employees tend to display higher levels of enthusiasm, enthusiasm, and energy when responding to and solving sustainability-related challenges. Furthermore, strategically recruiting employees with the requisite knowledge, experience, innovative spirit, and attitudes in line with the organization’s innovation strategies, systems, values, and goals may facilitate their participation in the company’s creative activities. Therefore, these employees show both in-role and extra-role contributions to team creative performance [12,63]. In an exploration of moderating mechanisms, the outcomes confirmed that the linkage between HIWP practice and team reflexivity was enhanced by the moderating effects of workload outcome (β =  0.18, p <  0.05) and physical work environment outcome (β =  0.13, p <  0.05) as an attribute of work demand. This highlights the notion that the level of employees showing higher engagement in organizational team reflexivity depends on the interplay of institutional factors (in this case, HIWP and work demand elements (workload and physical work environment). In essence, the different dimensions of teams, whether they are formed voluntarily or involuntarily, are contingent on the alignment between individual employees’ insights, values, customs, and norms and the organization’s procedures, goals, customs, and norms [17].

Thus, in the context of the service industry, the fusion of individual and team-related ecological factors (exemplified by the practice of HIWP) may catalyze to encourage employees to engage in team reflexivity [63]. This phenomenon can be attributed to the intertwining of participants’ cultural norms, beliefs, and values in their interpretation of role expectations and procedures, their adaptability to changing business needs, and their perceptions of HRM practices and organizational policy’s influence [64]. In simple words, an individual’s perception and understanding of an organization’s activities and efforts in the context of its surroundings can lead to differences in standards, civilizations, cultural norms, and principles. Overall, the outcomes of this study validate the proposed hypothesis, thereby contributing to the advancement of HIWP theory and practice as well as the literature on team creative performance.

### Theoretical contribution

In the organization’s context, this investigation is helpful to contribute to the key driving force promoting TCP. However, past literature ignored HIWPs’ significance in the scenario of team creativity performance (TCP). This study contributed by using the multilevel model of HIWPs which includes power, information, rewards, and knowledge. Most of the earlier studies consider it as a single level, for all individual, team, and organizational perspectives [23]. This study explored the relationship between HIWPs and team creative performance in a different way which has been done by very few studies at the individual level but not at team levels. Such relationships should be investigated as HIWP practices may be one of the most significant, expensive, and controllable in an organizational context.

Work demand as a moderator overcomes stress and enhances TCP. It also motivates HR practices to perform TCP [65]. Thus, the consequences are also reliable with previous learning, highlighting the need to decrease the work demand in the context of team performance. Further, the moderating relationship of workload between HIWPs and TR worked for all practices. The results are also justified from the previous literature [20]. It signals the importance of workload in the hotel industry and emphasizes that workload and schedules should be appropriately managed for hotel employees, otherwise, a high workload can decrease the creativity among different hotel teams [66]. Along with the workload, the physical work environment for the team is also a significant indicator to foster team performance. The interaction results found that a supportive physical work environment may increase the team’s creative performance. The results were in line with the previous studies as previous studies emphasized the importance of a supportive physical work environment in the hotel industry [48]. Hence, an important insight gained from this study is that team reflexivity can mediate more effectively between high-involvement work practice (HIWP) and team creative performance (TCP) than certain attitude constructs. Compared with emotional commitment and organizational citizenship behavior (OCB), team reflexivity has a broader scope and includes cognitive, affective, and behavioral dimensions. This wider range means that team members who exhibit higher engagement are likely to be more likely to exhibit diverse cognitive, attitudinal, and behavioral outcomes [22,23].

Another important insight gained from this study is the recognition that team reflexivity exhibits a multidimensional nature, manifesting itself through different mechanisms. In particular, this study highlights the synergistic impact of high-involvement work practice (HIWP) and work demand attributes on team reflexivity. The combination of institutional variables (represented by HIWP) and individual variables helps to expand the thrust of the CW theory. Moreover, CWT is favored because it provides a detailed, practical, and adaptable framework for understanding and managing cognitive demands in complex environments. Its emphasis on task complexity, practical applications in high-stress environments, comprehensive measurement techniques, and the integration of human factors make it an excellent choice for studying cognitive workload in specific professional contexts. The theory examines the relationship between task demands, cognitive resource capacity, and their impact on performance, attention, decision-making, and learning [28]. This investigation specifically delves into the combined interplay between HIWP practices (including power, information, reward, and knowledge) and specific work demand characteristics (including workload and physical work environment) that lead to increased team reflexivity. This means that implementing HIWP practices in the work environment alone may not promote team reflexivity; it is the assortment of contextual and personal variables that actively engage employees’ intention and willingness with the organizational relevance of the team. These results also claim that a high workload may decrease the innovative perspective among employees. These results verified the application of CWT in this context and are consistent with the earlier studies as work demand significantly creates a vital role in decreasing individual and TCP [65]. Team members should interact with each other to share their workload which could help supervisors and management to distribute the proper workload among teams and workers. Overall, the model provides an innovative perspective on the complexities of team creative performance, thereby paving the way for interesting avenues of future research.

### Practical implications

This research has multiple potential consequences for the service industry in Pakistan. The findings of the study recommend that companies can increase work creativity through HIWPs. Additionally, HRM practices are the most significant exerting influence on employees and teams within the organizations. From the multilevel model of HIWPs involving power, information, rewards, and knowledge, HRM managers should find out the relevancy of practice to increase employee or team performance. Changing business environments and cut-throat competition direct organizations to prioritize HIWPs to survive and focus on TCP [32]. The primary focus of the organizations should be team performance which enhances the creativity of employees, and the team performance should be based on high-involvement work practices (HIWPs) that are the main idea of this study. In the team creative performance (TCP) creativity obtains competitive benefits. This creativity should depend on both the individual and the team. HIWPs variable knowledge is considered as an insignificant predictor with TCP because organizations should arrange the training sections to extend the employees’ working knowledge and communication skills.

Moreover, organizations should carefully apply the HIWPs with increased TCP as the Asian countries had the conventional approach to power behavior and are disinclined to adopt the transformation of organizations. Secondly, this research found an important part of work demand among HIWPs and TCP. Companies should give special consideration to work demands while trying to boost creativity. Extensive duties, tight work schedules, supervisor’s strictness, and negative feedback may decrease teams’ creativity and push them to focus on current responsibilities rather than making them proactive. In a more specific context, this study provides evidence that recruiting individuals accustomed to increased workloads and demanding physical work environments increases the direct impact of high-involvement work practice (HIWP) interventions on team reflexivity. Furthermore, the mediating role played by team reflexivity provides insights into improving team creativity performance. This means that supervisors have the potential to encourage team reflexivity, thereby improving creative performance within teams and organizations. When supervisors create an environment that encourages team members to learn from mistakes, embrace team member diversity, and engage in thoughtful reflection, team members’ psychological safety is enhanced[29]. In turn, this is expected to enhance collective creativity within the team. Furthermore, given the importance of reflexivity in team learning and innovation, it was observed that the impact of team reflexivity on innovation is more pronounced in more demanding work environments [42], promoting team reflexivity is becoming increasingly important for modern work teams aiming to improve creative performance. While the moderating effect of the work demand with its elements WL and PWE in between HIWP and TR. Work demand with its elements WL and PWE as moderators overcomes stress and enhances TR. It also motivates HR practices to perform TCP [65]. Thus, the consequences are also reliable with previous studies, highlighting the need to decrease the work demand in the context of team performance. Along with the workload, PWE for the team is also a significant indicator of fostering team performance. The interaction results found that a supportive physical work environment may increase TCP. The results were in line with the previous studies which emphasized the importance of a supportive PWE in hotels [48]. These results also claim that a high workload may decrease the innovative perspective among employees. These results verified the application of CWT in this context and are consistent with the earlier studies as work demand significantly creates a vital role in decreasing individual and TCP [65]. Team members should interact with each other to share their workload which could help supervisors and management to distribute the proper workload among teams and employees.

## Limitations and future research direction

This investigation has a significant background other than having a specific limitation. Firstly, the focus of this study is only on the Pakistani hotel industry with a limited population. However, these findings may not apply directly to other sectors or regions because they offer valuable insights into the hospitality industry in a developing country. Secondly, the data in this study is cross-sectional. For the future view, longitudinal data can also be used for significant results. While this study represents a significant advance in the field of team creative performance (TCP) literature by delving into new research avenues, specifically investigating the role of team reflexivity in mediating the relationship between TCP and the synergistic effects of high-involvement work practices role (HIWP) and work demand attributes, it is necessary to examine these findings in the context of the inherent limitations present in this study. Further, the study model of this study introduced the moderating role of workload and physical work environment as well as TR as a mediator in the relationship of HIWPs, and TCP. However, this relationship can further be validated by introducing different mediators or moderators. However, other different variables can also consider mediating roles of different variables, e.g., internal social structure, knowledge management capacities, and collective efficacy[32]. Further, the present study recognized that the cultural context of Pakistan influenced the implementation and effects of HIWPs, potentially limiting the applicability of these findings to other cultural settings. Our study aimed to explore these practices within the specific context of Pakistan. Moreover, the HIWS principles (empowerment, collaboration, and continuous learning) are fundamental to many knowledge-based roles, it is possible to expand the usage of the given framework to IT and other businesses, as well as to finance, healthcare, and education services that appreciate flexibility and creativity.

For future work, contextual factors such as cultural values can also be used for moderating roles. Thus, several psychological or economic variables can also be used as moderators between HIWPs and TCP for future research. Finally, other studies can also analyze the results in the light of the recommendations of Khalilzadeh and Tasci [67] where a large sample size can be considered and can also report effect size measures such as Etta or Omega squared values for moderation effects, Cohen’s f-square for regression coefficients to obtain an in-depth view of results. Moreover, the present study acknowledges that incorporating qualitative research methods could provide deeper insights into the mechanisms of how HIWPs affect team creativity. However, the current study was designed to focus on quantitative analysis to assess these relationships at a broader level.

## Conclusion

Creativity is an important aspect of the service industry, especially in hotels where promoting creativity and innovation can bring fruitful results in organizational performance. However, hotels in developing countries especially in Pakistan do not consider contemporary management practices. Considering the scarcity of studies analyzing the role of HIWPs on TCP, this study aimed at analyzing the role of HIWPs enhancing TCP with the mediating role of TR as well as moderating the role of work demand attributes. Results found that the association between HIWPs and team creative performance is significant for team functioning, and this study found that HIWPs such as power and rewards are required and supportive when there is a tight work schedule. It is challenging for teams to effectively manage a physical work environment that cannot be alleviated by doing more and more work or following traditional work practices. It also validates the application of CWT in the team performance context. This study provides a way to cope with a challenge by introducing different practices to make teams more proactive, and sharper and to work smarter. Therefore, our findings recommend that improvement in the work environment is possible through HIWPs and innovation. Hotels can gain a competitive advantage by implementing HIWPs, however, significant training and workshops are necessary to promote the HIWPs.
